# The causal association between epilepsy and amyotrophic lateral sclerosis: A two‐sample Mendelian randomization study

**DOI:** 10.1002/brb3.70018

**Published:** 2024-09-27

**Authors:** Yayong Cui, Junyu Chen, Hong Li, Dong Zheng, Xiaolei Shi

**Affiliations:** ^1^ Department of Neurology, The Affiliated Brain Hospital Guangzhou Medical University Guangzhou China; ^2^ School of Mental Health Guangzhou Medical University Guangzhou China; ^3^ Institute of Psychiatry and Psychology Guangzhou Medical University Guangzhou China; ^4^ Key Laboratory of Neurogenetics and Channelopathies of Guangdong Province and the Ministry of Education of China Guangzhou Medical University Guangzhou China; ^5^ Guangdong Engineering Technology Research Center for Translational Medicine of Mental Disorders Guangzhou Medical University Guangzhou China; ^6^ Department of Neurosurgery, Nanfang Hospital Southern Medical University Guangzhou China; ^7^ Geriatric Neuroscience Center, The Affiliated Brain Hospital Guangzhou Medical University Guangzhou China

**Keywords:** amyotrophic lateral sclerosis, causal association, epilepsy, Mendelian randomization

## Abstract

**Objectives:**

Epilepsy and amyotrophic lateral sclerosis (ALS) are common neurological disorders. The association between the two disorders has been raised in observational studies. However, it is uncertain to what extent they have mutual causal effects. In this study, we aimed to investigate their causal association using a two‐sample Mendelian randomization (MR) method.

**Methods:**

We performed a two‐sample bidirectional MR analysis to evaluate the causal association of epilepsy with the risk of ALS. Publicly published genome‐wide association study statistics for epilepsy and ALS were used in the study. The primary analysis included genetic variants with a *p* value of less than 1 × 10^–5^ as instrumental variables. We applied several alternative methods, including inverse variance weighting, weighted median, simple mode, weighted mode, MR‐Egger regression and MR pleiotropy residual sum and outlier, and statistical graphs to assess the associations of epilepsy and its subtype with the risk of ALS. Reverse MR analyses were also performed to examine the association of ALS with the risk of epilepsy.

**Results:**

The primary MR analysis found no causal effect of epilepsy on risk of ALS (odds ration [OR]: 1.133, 95% confidence interval [CI]: 0.964–1.332, *p* = .130). Among subtypes of epilepsy, it also failed to observe any causal association between general epilepsy and ALS (OR: 1.036, 95% CI: 0.969–1.108, *P* = .300). However, focal epilepsy contributed to an increase in the risk of ALS (OR: 1.177, 95% CI: 1.027–1.348, *p* = .019). Moreover, the investigation of reverse causalities did not reveal significant results.

**Conclusions:**

The current study supports a causal influence of focal epilepsy on ALS risk. Future studies are needed to explore its potential role in ALS.

## Graphical abstract

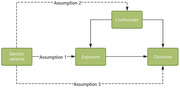

## 1 INTRODUCTION

Amyotrophic lateral sclerosis (ALS) is a progressing neurological disorder, characterized by the degeneration of motor neurons, with ∼50% of cases experiencing cognitive and behavioral symptoms (Masrori & Van Damme, 2020). Abnormal patterns of neuron excitability and synaptic activities have been observed in ALS (Fogarty, 2018). It is believed that altered integration of neurons and synapses is responsible for the onset and progression of ALS (Fogarty, 2018).

Epilepsy is one of the most common neurological disorders, affecting over 50 million people all over the world (Singh & Sander, 2020). Excessive neuronal activity can result in repeated and unpredictable epileptic seizures, inducing cognitive impairment and mental instability, and even life‐threatening events (Giourou et al., 2015). Studies have reported the incidence of epileptic seizures in ALS patients (Dag et al., 2014), indicating the potential association between the two disorders. The hyperactivities of neuronal circuit, for some extent, explain the link between epilepsy and ALS. However, it is still unknown whether their relationship has a causal nature.

Mendelian randomization (MR) helps clarify the causal relationship between exposure and outcome, avoiding potential associative inferences (Richmond & Smith, 2022). Genetic variation is used as an instrumental variable to deduce the causal relationship between exposure and outcome, which will not be affected by acquired environmental factors, lifestyles, behavioral changes, and other confounding factors (Richmond & Smith, 2022). It meets the requirement of time sequence in the determination of causality, which can effectively avoid the interference of reverse causation. Therefore, we applied a two‐sample MR to explore the causal relationship between epilepsy and ALS.

## 2 METHODS

### 2.1 Study design

**FIGURE 1:**
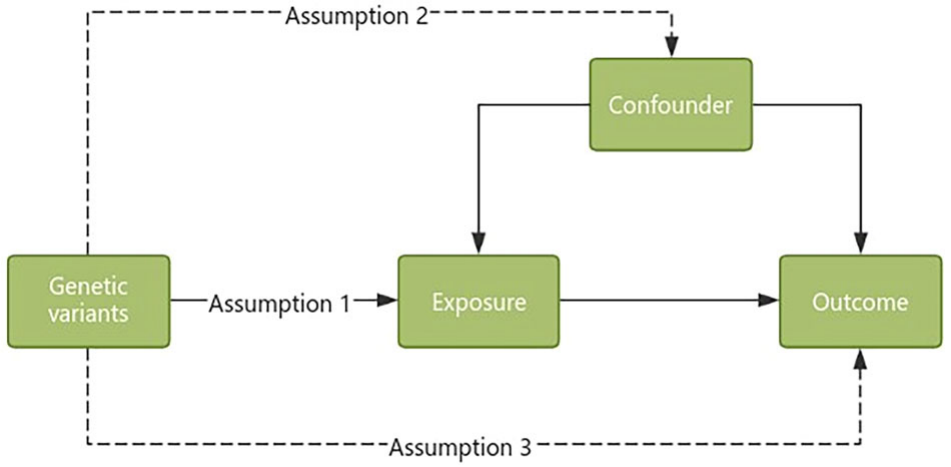
The design of the MR study. Assumption 1, the genetic variation used as an instrumental variable should be closely associated with the exposure. Assumption 2, the genetic variation should not be associated with any confounding factors. Assumption 3, the selected genetic variation should affect the risk of the outcome only through the risk factor, rather than through other pathways. MR, Mendelian randomization.

In the present study, we performed a bidirectional two‐sample MR analysis to estimate the causal associations between epilepsy (all forms of epilepsy, generalized epilepsy, focal epilepsy) and ALS. MR analysis used genetic variants as instrumental variables to investigate the relationship between certain exposure and outcome. As depicted in Figure 1, there were three key assumptions of MR framework: (1) genetic variants proposed as instrumental variables (IVs) should be robustly associated with the exposure; (2) genetic variants used as IVs should not be associated with any confounders; and (3) the selected genetic variants should affect the risk of the outcome only through the risk factor, but not via alternative pathways. Genome‐wide association study (GWAS) summary statistics were searched to extract leading single nucleotide polymorphisms (SNPs) associated with epilepsy or ALS as genetic instrumental variables. The forward MR analyses considered epilepsy as the exposure and ALS as the outcome, while the reverse MR raised ALS as the exposure and epilepsy as the outcome. This study was based on publicly available summary statistics, and no ethical approval was required.

### 2.2 Data sources

Genetic instruments for epilepsy were obtained from a large‐scale GWAS of European ancestry, including 69,995 cases of all forms of epilepsy, with 3024 cases affected by generalized epilepsy, 3688 cases affected by focal epilepsy from the study by International League Against Epilepsy Consortium on Complex Epilepsies (2023). Genetic variants for ALS were obtained from a large‐scale GWAS by Nicolas et al. (2018), including 20,806 ALS cases and 59,804 controls. The participants in these datasets above were of European ancestry.

### 2.3 Selection of instruments

We set a relatively relaxed criteria to 1 × 10^−5^ for IVs selection from GWAS data of epilepsy in the primary analysis (Table S1, epilepsy; Table S2, focal epilepsy; Table S3, generalized epilepsy). We performed the exclusion of SNPs in linkage disequilibrium, using the criteria of an *R* ^2^ value of less than 0.001 and a genetic distance of 10,000 KB. Genetic variants were screened at the threshold of genome‐wide significance at 5 × 10^−8^ for additional analysis as a supplemental analysis (Tables S1–S3). Primary and supplemental analyses were also done in the reverse analysis of the effects of ALS on epilepsy and its subtypes. IVs for ALS can be found in Table S4.

GWAS Catalog (https://www.ebi.ac.uk/gwas) and NCBI SNP database (https://www.ncbi.nlm.nih.gov/snp) were searched for the related traits and genes associated with ALS, based on the chosen IVs of epilepsy and its subtypes. The corresponding SNPs were deleted from the respective analysis (Table S5).

### 2.4 Statistical analysis

In our main analysis, we applied the inverse‐variance weighted (IVW) approach to combine the effect estimates from all IVs to acquire the causal analysis. In addition, we performed sensitivity analyses, including weighted median, simple mode, weighted mode, MR‐Egger regression, and MR pleiotropy residual sum and outlier (MR‐PRESSO), to assess pleiotropy and potential genetic outliers. Assuming that at least 50% of the SNPs are valid, the weighted median method can generate consistent causal estimates. The MR‐Egger regression can detect and correct for possible pleiotropy, and the *p* value of the intercept > .05 indicates no horizontal pleiotropic effects. For the exclusion restriction assumption (Richmond & Smith, 2022), the MR‐Egger regression intercept and its 95% confidence intervals (CIs) were used to investigate the degree of bias in casual estimates due to directional pleiotropy. Furthermore, the MR‐PRESSO method was applied to detect significant outliers and correct for horizontal pleiotropic effects through outlier removal. The MR‐PRESSO global test evaluates whether horizontal pleiotropy among all instruments was present. Cochran's *Q* statistic and leave‐one‐out analysis were performed to evaluate the degree of heterogeneity across each SNP. The MR‐Egger intercept test, the MR‐PRESSO global test, and visual inspection of the funnel plot were conducted to detect horizontal pleiotropy. The *p* value < .05 indicated that the IVW results might be invalid due to horizontal pleiotropy. All analyses were two‐sided and performed using the TwoSampleMR packages in the R software (version 4.1.3).

## 3 RESULT

### 3.1 The effects of epilepsy on ALS

**FIGURE 2:**
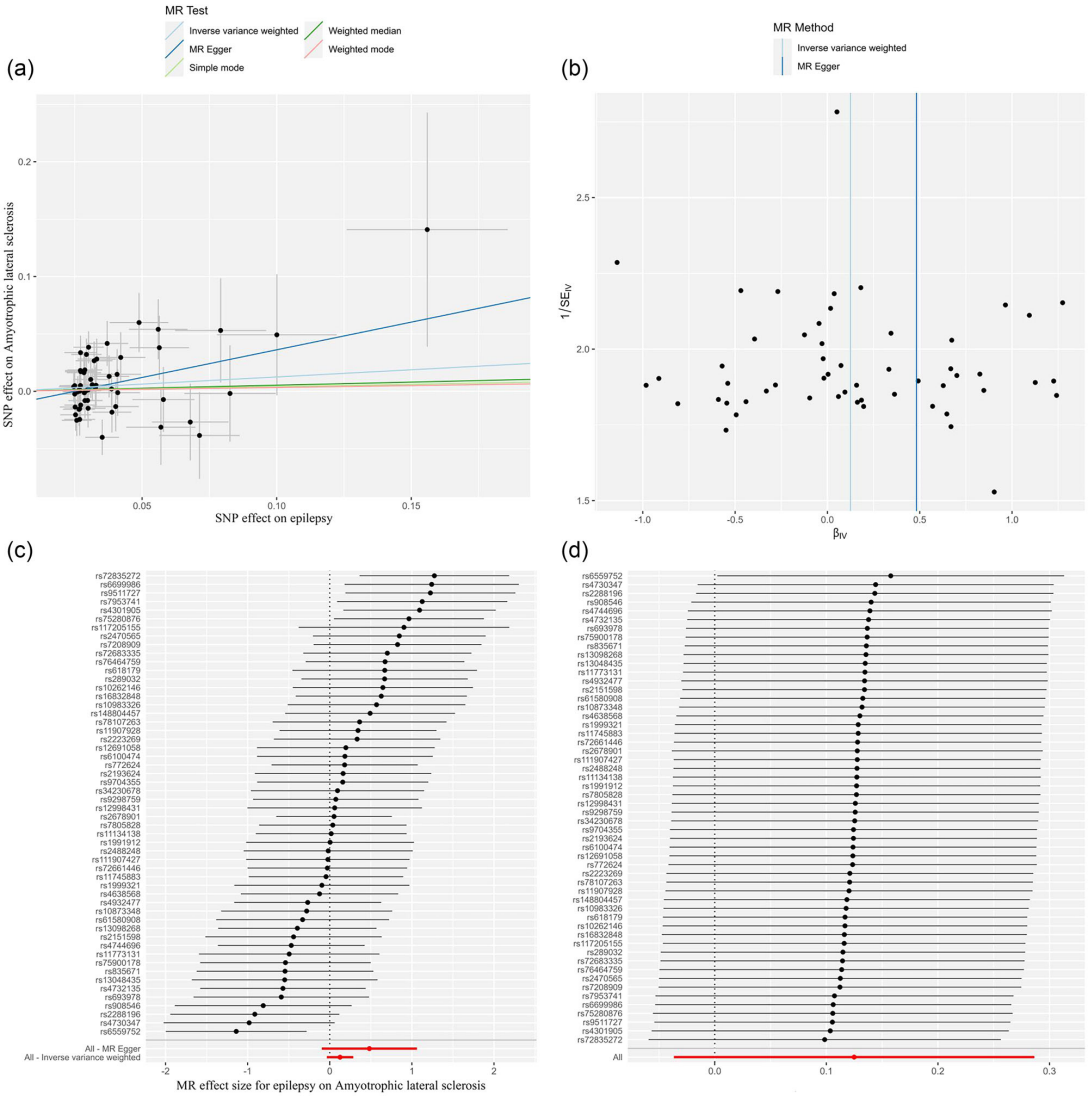
The causal effect of epilepsy on ALS risk in primary analysis. (a) Scatter plot, (b) funnel plot, (c) forest plot, and (d) leave‐one‐out plot. ALS, amyotrophic lateral sclerosis; MR, Mendelian randomization; SNP, single nucleotide polymorphism.

Under the IVW model in the primary analysis (*p* < 1 × 10^−5^ for IVs selection), we did not find evidence of the association of epilepsy with the risk of ALS (odds ratio [OR]: 1.133, 95% CI: 0.964–1.332, *p* = .130, Table 1). As shown in Figure 2, the scatter plot and forest plot visually showed the relationship between epilepsy and ALS risk. The results of Cochran's Q test, MR‐Egger intercept test, and MR‐PRESSO global test are displayed in Table 2. For SNP integration, we conducted the leave‐one‐out analysis and generated forest plots (Figure 2).

**TABLE 1:** Mendelian randomization (MR) analysis of the associations between epilepsy and amyotrophic lateral sclerosis (ALS) in primary and supplemental analysis.

	Exposure	Method	nSNP	OR	(95% CI)	*p* value
Primary analysis						
	Epilepsy	IVW	54	1.133	0.964–1.332	.130
		Weighted median	54	1.055	0.856–1.299	.616
		MR Egger	54	1.620	0.907–2.882	.109
		Simple mode	54	1.042	0.610–1.777	.882
		Weighted mode	54	1.034	0.660–1.620	.885
	Generalized epilepsy	IVW	72	1.036	0.969–1.108	.300
		Weighted median	72	1.059	0.956–1.173	.275
		MR Egger	72	0.900	0.692–1.171	.435
		Simple mode	72	1.146	0.907–1.446	.257
		Weighted mode	72	1.155	0.928–1.438	.202
	Focal epilepsy	IVW	45	1.177	1.027–1.348	.019^a^
		Weighted median	45	1.058	0.886–1.265	.533
		MR Egger	45	0.951	0.554–1.633	.855
		Simple mode	45	1.062	0.697–1.619	.781
		Weighted mode	45	1.055	0.711–1.567	.791
Supplemental analysis						
	Epilepsy	IVW	2	0.652	0.207–2.048	.464
	Generalized epilepsy	IVW	18	1.055	0.918–1.213	.447
		Weighted median	12	1.064	0.896–1.265	.479
		MR Egger	12	0.785	0.411–1.500	.481
		Simple mode	12	1.185	0.877–1.602	.292
		Weighted mode	12	1.185	0.872–1.612	0.302
	Focal epilepsy	–	0	–	–	–

Note: CI, confidence interval; IVW, inverse‐variance weighted; nSNPs, number of single nucleotide polymorphisms; OR, odds ratio.

^a^ Indicates statistical significance at *p* < .05.

**TABLE 2:** Heterogeneity and pleiotropy tests for the associations between amyotrophic lateral sclerosis (ALS) and epilepsy in primary and supplemental analysis.

	Cochrane's *Q* test		MR‐Egger intercept test		MR‐PRESSO global test
	*Q*‐value	*P*_Q_	Intercept	*P*_intercept_	*p* value
Primary analysis					
Epilepsy	74.345	0.028^*^	−0.012	0.214	.065
Focal epilepsy	51.198	0.209	0.009	0.429	.224
Generalized epilepsy	70.250	0.503	0.008	0.281	.426
Supplemental analysis					
Epilepsy	–	–	–	–	–
Generalized epilepsy	12.575	0.322	0.021	0.381	.152
Focal epilepsy	–	–	–	–	–

Abbreviation: MR‐PRESSO: Mendelian randomization‐Pleiotropy Residual Sum and Outlier.

^*^ indicated statistically significance P < .05.

**FIGURE 3:**
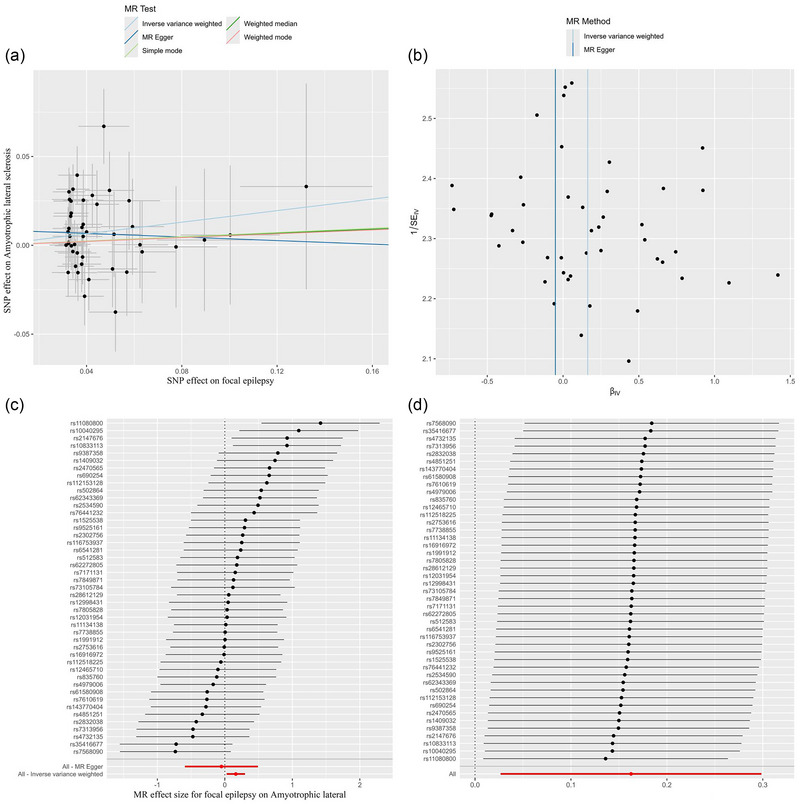
The causal effect of focal epilepsy on ALS risk in primary analysis. (a) Scatter plot, (b) funnel plot, (c) forest plot, and (d) leave‐one‐out plot. ALS, amyotrophic lateral sclerosis; MR, Mendelian randomization.

**FIGURE 4:**
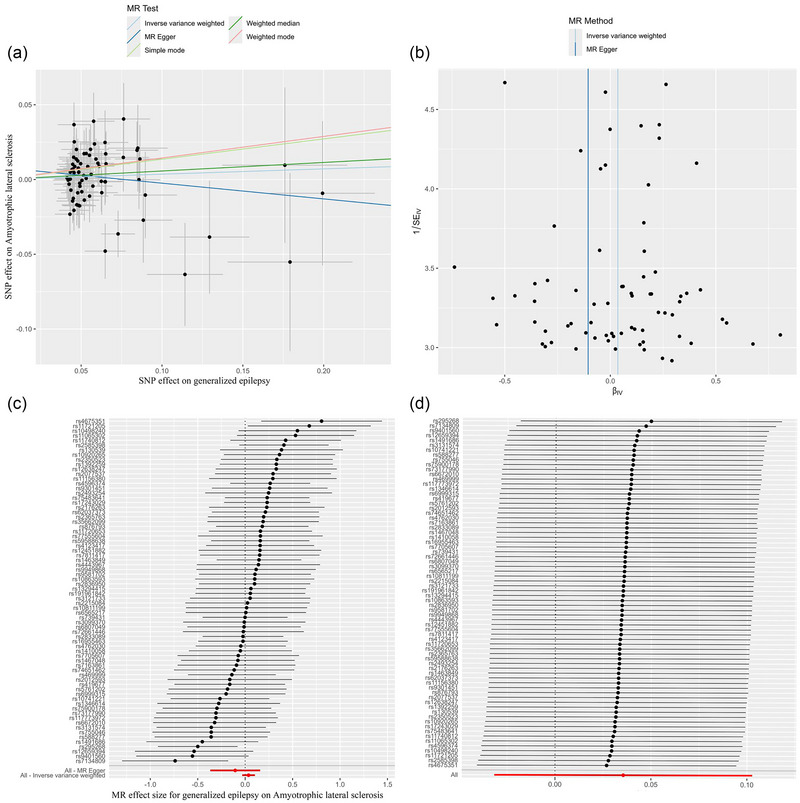
The causal effect of generalized epilepsy on ALS risk in primary analysis. (a) Scatter plot, (b) funnel plot, (c) forest plot, and (d) leave‐one‐out plot. ALS, amyotrophic lateral sclerosis; MR, Mendelian randomization.

We further conducted the analysis based on subtypes of epilepsy. As shown in Table 1, the IVW model showed a statistically significant association between focal epilepsy (per SD increase) and risk of ALS (OR: 1.177, 95% CI: 1.027–1.348, *p* = .019, Table 1, Figure 2). No evidence of heterogeneity or directional pleiotropy was found in the analysis of focal epilepsy (both *p* values > .1, Table 2). We also applied the leave‐one‐out analysis and identified no SNPs that would significantly affect IVW estimate (Figure 3). In addition, no significant association was found for generalized epilepsy with ALS (OR: 1.036, 95% CI: 0.969–1.108, *p* = 0.300, Figure 4).

Moreover, supplemental analysis was done based on the IVs selection criteria of *p* < 5 × 10^−8^ (Table 1). No significance was found between epilepsy and risk of ALS, with only two SNPs included (OR: 0.652, 95% CI: 0.207–2.048, *p* = 0.464). Null association was indicated for the causal effects of generalized epilepsy on ALS (OR: 1. 055, 95% CI: 0.918–1.213, *p* = 0.447) (Figure S1). Moreover, it failed to perform the analysis of focal epilepsy on ALS for the reason that no SNPs were found at the significance threshold.

### 3.2 The effects of ALS on epilepsy

The reverse MR analysis (Table S6) suggested that neither epilepsy (Figure S2) nor subtypes of epilepsy (focal epilepsy, Figure S3; generalized epilepsy, Figure S4) were causally associated with ALS.

## 4 DISCUSSION

This study investigated the causal associations of genetically predicted epilepsy and its subtypes with ALS using a two‐sample MR approach. It indicated that focal epilepsy was causally linked with ALS. No causality was found for all forms of epilepsy with ALS, as well as between generalized epilepsy and ALS.

Currently, limited evidence could be found regarding the association between epilepsy and ALS. A post‐GWAS study by Schijven et al. (2020) explored the correlation between the two disorders, and subgroup analysis was performed based on epileptic forms. Negative results were found between the two disorders, which is consistent with our findings except for the causal association between focal epilepsy and ALS revealed by our study. This could be attributed to the different GWAS data sources. First, although most individuals in the study by Schijven et al. (2020) were of European ancestry, there were still some individuals of African and Asian ancestry in the epilepsy GWAS. However, we used a relatively new GWAS dataset of epilepsy (International League Against Epilepsy Consortium on Complex Epilepsies, 2023) in which all the cases were from European population. Moreover, the GWAS data for ALS (Nicolas et al., 2018) in our study were quite different with a larger dataset, covering 20,806 cases and 59,804 controls. The previous study used data from van Rheenen et al. (2016), which included 12,577 cases and 23,475 controls. The larger sample size, with all cases from the same ancestry, ensured the credibility of our findings.

Furthermore, we validated the causal association between focal epilepsy and ALS, but not between generalized epilepsy and ALS. Generally, according to ictal symptoms, epilepsy could be classified into two major subgroups: generalized and focal (partial, local) epilepsy (Fisher, 2017). Focal epilepsy comprises localized areas of abnormal electrical activity which could spread to contiguous and non‐contiguous areas (Götz‐Trabert et al., 2008; Turkdogan et al., 2005). Hyperexcitability in local seizure onset area may be due to a variety of factors, such as the presence of pathological tissue, downregulation of local inhibitory circuits, or upregulation of excitatory circuits (Azimzadeh & Beheshti, 2023; Iizuka et al., 2002; Navidhamidi et al., 2017). This echoes to the pathological features of ALS. ALS also represents a focal onset form, occurring at an apparently random location, and progressing along human body (Ravits, 2014). Therefore, the focal changes in specific region in central nervous system may underlie the link between focal epilepsy and ALS. Studies have indicated that changes of ion channels, for example, Na+ and K+ channels, are involved in the development of focal epileptic occurrence (Brines et al., 1995). They are responsible for muscle bundle tremors and muscle spasms in ALS patients (Tarantino et al., 2022). Therapeutic agents, retigabine (regulating sodium and calcium currents) (Czuczwar et al., 2010) and riluzole (modulating sodium channels) (Urbani & Belluzzi, 2000), are effective in both epilepsy and ALS. This also pointed the similar therapeutic options of ALS and focal epilepsy.

A key strength of the study is that we examined the genetic causal effects of epilepsy subtypes on ALS by exploring data from relatively large GWAS sources. Moreover, we addressed the analysis of horizontal pleiotropy by performing MR‐PRESSO global test and MR‐Egger intercept test, and by the visual inspection of funnel plot, indicating no evidence of horizontal pleiotropic effects. The robustness of the significant findings was further validated by detecting heterogeneity using Cochrane's Q test and leave‐one‐out sensitivity analysis.

Indeed, our research also has some limitations. The findings were derived from GWAS datasets of European ancestry. Whether the results could be applied in the general population remains to be verified. The study only used the MR approach to assess the correlation between epilepsy and ALS. More post‐GWAS methods should be available in further analysis.

## 5 CONCLUSION

The study provides evidence of causal influence of focal epilepsy on ALS risk. A new light is cast on the potential role of epilepsy in ALS. Future studies are needed to explore the underlying mechanism.

## AUTHOR CONTRIBUTIONS

**Yayong Cui**: Writing—original draft; Data curation; Formal analysis. **Junyu Chen**: Writing—original draft; Data curation; Formal analysis. **Hong Li**: Formal analysis; Data curation. **Dong Zheng**: Conceptualization; Supervision; Funding acquisition. **Xiaolei Shi**: Conceptualization; Supervision; Funding acquisition.

### PEER REVIEW

The peer review history for this article is available at https://publons.com/publon/10.1002/brb3.70018.

## Supporting information

Supporting Information

## Data Availability

The data that support the findings of this study are available in the supplementary material of this article.

## References

[brb370018-bib-0001] Azimzadeh, M. , & Beheshti, S. (2023). Down regulation of the hippocampal ghrelin receptor type‐1a during electrical kindling‐induced epileptogenesis. Epilepsy Research, 189, 107064. 36516566 10.1016/j.eplepsyres.2022.107064

[brb370018-bib-0002] Brines, M. L. , Tabuteau, H. , Sundaresan, S. , Kim, J. , Spencer, D. D. , & de Lanerolle, N. (1995). Regional distributions of hippocampal Na^+^, K^+^‐ATPase, cytochrome oxidase, and total protein in temporal lobe epilepsy. Epilepsia, 36, 371–383. 7607116 10.1111/j.1528-1157.1995.tb01012.x

[brb370018-bib-0003] Czuczwar, P. , Wojtak, A. , Cioczek‐Czuczwar, A. , Parada‐Turska, J. , Maciejewski, R. , & Czuczwar, S. J. (2010). Retigabine: The newer potential antiepileptic drug. Pharmacological Reports, 62, 211–219. 20508276 10.1016/s1734-1140(10)70260-7

[brb370018-bib-0004] Dag, E. , Sahin, O. , Gökçe, B. , & Erdemoglu, A. K. (2014). Status epilepticus in a patient with amyotrophic lateral sclerosis/Amiyotrofik Lateral Sklerozlu Bir Hastada Status Epileptikus. Journal of Emergency Medicine Case Reports, 5, 5–7.

[brb370018-bib-0005] Fisher, R. S. (2017). The new classification of seizures by the international league against epilepsy 2017. Current Neurology and Neuroscience Reports, 17, 48. 28425015 10.1007/s11910-017-0758-6

[brb370018-bib-0006] Fogarty, M. J. (2018). Driven to decay: Excitability and synaptic abnormalities in amyotrophic lateral sclerosis. Brain Research Bulletin, 140, 318–333. 29870780 10.1016/j.brainresbull.2018.05.023

[brb370018-bib-0007] Giourou, E. , Stavropoulou‐Deli, A. , Giannakopoulou, A. , Kostopoulos, G. K. , & Koutroumanidis, M. (2015). Introduction to epilepsy and related brain disorders. In N. Voros & C. Antonopoulos (Eds.), Cyberphysical systems for epilepsy and related brain disorders: Multi‐parametric monitoring and analysis for diagnosis and optimal disease management (pp. 11–38). Springer.

[brb370018-bib-0008] Götz‐Trabert, K. , Hauck, C. , Wagner, K. , Fauser, S. , & Schulze‐Bonhage, A. (2008). Spread of ictal activity in focal epilepsy. Epilepsia, 49, 1594–1601. 18435751 10.1111/j.1528-1167.2008.01627.x

[brb370018-bib-0009] Iizuka, T. , Sakai, F. , Suzuki, N. , Hata, T. , Tsukahara, S. , Fukuda, M. , & Takiyama, Y. (2002). Neuronal hyperexcitability in stroke‐like episodes of MELAS syndrome. Neurology, 59, 816–824. 12297560 10.1212/wnl.59.6.816

[brb370018-bib-0010] International League Against Epilepsy Consortium on Complex Epilepsies . (2023). GWAS meta‐analysis of over 29,000 people with epilepsy identifies 26 risk loci and subtype‐specific genetic architecture. Nature Genetics, 55, 1471–1482. 37653029 10.1038/s41588-023-01485-w PMC10484785

[brb370018-bib-0011] Masrori, P. , & Van Damme, P. (2020). Amyotrophic lateral sclerosis: A clinical review. European Journal of Neurology, 27, 1918–1929. 32526057 10.1111/ene.14393 PMC7540334

[brb370018-bib-0012] Navidhamidi, M. , Ghasemi, M. , & Mehranfard, N. (2017). Epilepsy‐associated alterations in hippocampal excitability. Reviews in the Neurosciences, 28, 307–334. 28099137 10.1515/revneuro-2016-0059

[brb370018-bib-0013] Nicolas, A. , Kenna, K. P. , Renton, A. E. , Ticozzi, N. , Faghri, F. , Chia, R. , Dominov, J. A. , Kenna, B. J. , Nalls, M. A. , Keagle, P. , & Rivera, A. M. (2018). Genome‐wide Analyses Identify KIF5A as a Novel ALS Gene. Neuron, 97, 1268–1283.e1266. 10.1016/j.neuron.2018.02.027 PMC5867896 29566793

[brb370018-bib-0014] Ravits, J. (2014). Focality, stochasticity and neuroanatomic propagation in ALS pathogenesis. Experimental Neurology, 262, 121–126. 25108067 10.1016/j.expneurol.2014.07.021

[brb370018-bib-0015] Richmond, R. C. , & Smith, G. D. (2022). Mendelian randomization: Concepts and scope. Cold Spring Harbor Perspectives in Medicine, 12, a040501. 34426474 10.1101/cshperspect.a040501 PMC8725623

[brb370018-bib-0016] Schijven, D. , Stevelink, R. , McCormack, M. , van Rheenen, W. , Luykx, J. J. , Koeleman, B. P. C. , Veldink, J. , & Catarino, C. B. (2020). Analysis of shared common genetic risk between amyotrophic lateral sclerosis and epilepsy. Neurobiology of Aging, 92, 153.e151–153.e155. 10.1016/j.neurobiolaging.2020.04.011 PMC7818383 32409253

[brb370018-bib-0017] Singh, G. , & Sander, J. W. (2020). The global burden of epilepsy report: Implications for low‐and middle‐income countries. Epilepsy & Behavior, 105, 106949. 32088583 10.1016/j.yebeh.2020.106949

[brb370018-bib-0018] Tarantino, N. , Canfora, I. , Camerino, G. M. , & Pierno, S. (2022). Therapeutic targets in amyotrophic lateral sclerosis: Focus on ion channels and skeletal muscle. Cells, 11, 415. 35159225 10.3390/cells11030415 PMC8834084

[brb370018-bib-0019] Turkdogan, D. , Duchowny, M. , Resnick, T. , & Jayakar, P. (2005). Subdural EEG patterns in children with taylor‐type cortical dysplasia: Comparison with nondysplastic lesions. Journal of Clinical Neurophysiology, 22, 37–42. 15689711 10.1097/01.wnp.0000150887.61562.26

[brb370018-bib-0020] Urbani, A. , & Belluzzi, O. (2000). Riluzole inhibits the persistent sodium current in mammalian CNS neurons. European Journal of Neuroscience, 12, 3567–3574. 11029626 10.1046/j.1460-9568.2000.00242.x

[brb370018-bib-0021] van Rheenen, W. , Shatunov, A. , Dekker, A. M. , McLaughlin, R. L. , Diekstra, F. P. , Pulit, S. L. , Van Der Spek, R. A. , Võsa, U. , De Jong, S. , Robinson, M. R. , & Yang, J. (2016). Genome‐wide association analyses identify new risk variants and the genetic architecture of amyotrophic lateral sclerosis. Nature Genetics, 48, 1043–1048. 27455348 10.1038/ng.3622 PMC5556360

