# Supplementary material for: The causal association between epilepsy and amyotrophic lateral sclerosis: A two‐sample Mendelian randomization study
Source: Brain Behav. 2024 Sep 27;14(10):e70018. doi: 10.1002/brb3.70018 (PMC11427789; doi:10.1002/brb3.70018)
Supplement: Supplementary file 1 — Supporting Information [file BRB3-14-e70018-s001.docx]

**Supplemental Table**

**Table S1.** Characteristics of selected SNPs for epilepsy.

| **SNP** | **Trait** | **Chr** | **Pos.** | **Effect allele** | **Other allele** | **EAF** | **Beta** | **SE** | ***P value*** | **R^2^** | **F statistic** |
| --- | --- | --- | --- | --- | --- | --- | --- | --- | --- | --- | --- |
| rs72661446 | epilepsy | 1 | 23740708 | A | C | 0.1306 | -0.04097 | 0.008305 | 8.09E-07 | 0.000381 | 24.33372 |
| rs6699986 | epilepsy | 1 | 7849656 | A | G | 0.696 | 0.027153 | 0.00603 | 6.71E-06 | 0.000312 | 20.27639 |
| rs835671 | epilepsy | 1 | 2.27E+08 | T | C | 0.4048 | -0.02514 | 0.005653 | 8.73E-06 | 0.000305 | 19.77527 |
| rs2488248 | epilepsy | 1 | 2.08E+08 | T | C | 0.4341 | 0.026085 | 0.005599 | 3.17E-06 | 0.000334 | 21.70567 |
| rs111907427 | epilepsy | 2 | 814643 | A | T | 0.0286 | 0.082672 | 0.016948 | 1.07E-06 | 0.00038 | 23.79413 |
| rs2678901 | epilepsy | 2 | 58138651 | A | G | 0.4048 | 0.038679 | 0.005647 | 7.41E-12 | 0.000721 | 46.90738 |
| rs60055328 | epilepsy | 2 | 1.67E+08 | T | C | 0.7601 | 0.04506 | 0.006501 | 4.19E-12 | 0.00074 | 48.03731 |
| rs12998431 | epilepsy | 2 | 1.9E+08 | A | G | 0.3432 | 0.026731 | 0.005907 | 6.03E-06 | 0.000322 | 20.47497 |
| rs2193624 | epilepsy | 2 | 57016392 | A | G | 0.4801 | -0.02463 | 0.00555 | 9.06E-06 | 0.000303 | 19.69526 |
| rs908546 | epilepsy | 2 | 1.13E+08 | C | G | 0.4116 | 0.025298 | 0.00568 | 8.42E-06 | 0.00031 | 19.83755 |
| rs2288196 | epilepsy | 2 | 1.52E+08 | A | G | 0.62 | -0.02683 | 0.005717 | 2.69E-06 | 0.000339 | 22.03299 |
| rs16832848 | epilepsy | 2 | 1.92E+08 | A | G | 0.6604 | -0.02725 | 0.005869 | 3.42E-06 | 0.000333 | 21.56607 |
| rs1991912 | epilepsy | 2 | 80799512 | T | C | 0.3879 | 0.026838 | 0.005703 | 2.52E-06 | 0.000342 | 22.14569 |
| rs75900178 | epilepsy | 2 | 1.21E+08 | T | C | 0.0381 | 0.071331 | 0.014964 | 1.87E-06 | 0.000373 | 22.72353 |
| rs9817162 | epilepsy | 3 | 1.15E+08 | T | C | 0.5848 | -0.02639 | 0.005648 | 2.98E-06 | 0.000338 | 21.82686 |
| rs2470565 | epilepsy | 3 | 11919651 | T | C | 0.192 | 0.033176 | 0.007083 | 2.81E-06 | 0.000341 | 21.93922 |
| rs772624 | epilepsy | 3 | 36299291 | T | G | 0.3447 | -0.0315 | 0.005836 | 6.79E-08 | 0.000448 | 29.12671 |
| rs13098268 | epilepsy | 3 | 30368470 | A | G | 0.0408 | -0.06791 | 0.014237 | 1.85E-06 | 0.000361 | 22.7522 |
| rs693978 | epilepsy | 4 | 38180161 | A | G | 0.6809 | -0.02659 | 0.005946 | 7.74E-06 | 0.000307 | 19.9982 |
| rs289032 | epilepsy | 5 | 1.14E+08 | A | G | 0.383 | 0.027092 | 0.005706 | 2.05E-06 | 0.000347 | 22.54276 |
| rs10078040 | epilepsy | 5 | 1.37E+08 | C | G | 0.4893 | -0.02482 | 0.005547 | 7.64E-06 | 0.000308 | 20.02508 |
| rs148804457 | epilepsy | 5 | 1.54E+08 | A | G | 0.0164 | 0.100059 | 0.022235 | 6.79E-06 | 0.000323 | 20.24934 |
| rs11134138 | epilepsy | 5 | 6093701 | T | C | 0.4552 | -0.02924 | 0.005575 | 1.57E-07 | 0.000424 | 27.50909 |
| rs11745883 | epilepsy | 5 | 73111953 | A | G | 0.569 | -0.02856 | 0.005603 | 3.46E-07 | 0.0004 | 25.97864 |
| rs7805828 | epilepsy | 7 | 22758562 | A | G | 0.4097 | 0.029906 | 0.005634 | 1.11E-07 | 0.000433 | 28.17409 |
| rs6960173 | epilepsy | 7 | 92991949 | C | G | 0.4301 | 0.025903 | 0.005645 | 4.45E-06 | 0.000329 | 21.05825 |
| rs11773131 | epilepsy | 7 | 66513939 | A | T | 0.244 | 0.029958 | 0.006507 | 4.14E-06 | 0.000331 | 21.19618 |
| rs4730347 | epilepsy | 7 | 78138925 | A | G | 0.4696 | 0.025765 | 0.005571 | 3.75E-06 | 0.000331 | 21.38989 |
| rs4732135 | epilepsy | 7 | 1.35E+08 | A | G | 0.4294 | -0.02683 | 0.005621 | 1.81E-06 | 0.000353 | 22.78091 |
| rs10262146 | epilepsy | 7 | 1.46E+08 | A | T | 0.7536 | -0.02875 | 0.006449 | 8.23E-06 | 0.000307 | 19.88213 |
| rs34230678 | epilepsy | 8 | 57083117 | T | C | 0.8168 | 0.032705 | 0.007186 | 5.33E-06 | 0.00032 | 20.711 |
| rs78107263 | epilepsy | 8 | 89397557 | A | G | 0.1091 | 0.040723 | 0.008895 | 4.70E-06 | 0.000322 | 20.95742 |
| rs117205155 | epilepsy | 8 | 10981008 | A | C | 0.0094 | 0.155924 | 0.029922 | 1.88E-07 | 0.000453 | 27.15369 |
| rs4744696 | epilepsy | 9 | 76435220 | A | G | 0.8198 | -0.03882 | 0.007219 | 7.56E-08 | 0.000445 | 28.91126 |
| rs9298759 | epilepsy | 9 | 16375403 | A | G | 0.2843 | 0.029967 | 0.006189 | 1.28E-06 | 0.000365 | 23.44423 |
| rs10983326 | epilepsy | 9 | 1.19E+08 | A | C | 0.2521 | 0.028439 | 0.006385 | 8.43E-06 | 0.000305 | 19.83755 |
| rs6559752 | epilepsy | 9 | 86570075 | T | C | 0.267 | -0.0352 | 0.006271 | 1.98E-08 | 0.000485 | 31.51605 |
| rs2151598 | epilepsy | 9 | 1.03E+08 | A | G | 0.3012 | -0.02722 | 0.006064 | 7.18E-06 | 0.000312 | 20.1415 |
| rs3740422 | epilepsy | 10 | 1.04E+08 | C | G | 0.3455 | 0.035111 | 0.005839 | 1.82E-09 | 0.000558 | 36.15497 |
| rs9704355 | epilepsy | 11 | 1.25E+08 | C | G | 0.8137 | -0.03292 | 0.007121 | 3.80E-06 | 0.000328 | 21.36219 |
| rs7953741 | epilepsy | 12 | 1.21E+08 | A | G | 0.8523 | 0.03704 | 0.007821 | 2.18E-06 | 0.000345 | 22.42898 |
| rs618179 | epilepsy | 13 | 44442033 | A | G | 0.0295 | -0.07918 | 0.016912 | 2.84E-06 | 0.000359 | 21.92039 |
| rs9511727 | epilepsy | 13 | 25824563 | T | C | 0.9302 | -0.04888 | 0.010906 | 7.38E-06 | 0.00031 | 20.08773 |
| rs4301905 | epilepsy | 13 | 53167513 | T | C | 0.4103 | -0.02935 | 0.005638 | 1.93E-07 | 0.000417 | 27.10168 |
| rs1950182 | epilepsy | 14 | 86055554 | C | G | 0.471 | 0.026491 | 0.005615 | 2.38E-06 | 0.00035 | 22.25884 |
| rs10873348 | epilepsy | 14 | 83710088 | T | G | 0.7199 | 0.028788 | 0.006178 | 3.17E-06 | 0.000334 | 21.71497 |
| rs72683335 | epilepsy | 14 | 80351333 | T | G | 0.1022 | 0.042083 | 0.009153 | 4.26E-06 | 0.000325 | 21.14098 |
| rs4932477 | epilepsy | 15 | 89702298 | T | C | 0.5264 | -0.02978 | 0.00558 | 9.40E-08 | 0.000442 | 28.49345 |
| rs4638568 | epilepsy | 16 | 50045839 | A | G | 0.0625 | -0.05793 | 0.011534 | 5.08E-07 | 0.000393 | 25.22978 |
| rs61580908 | epilepsy | 16 | 77334604 | T | C | 0.8808 | 0.040211 | 0.008592 | 2.87E-06 | 0.00034 | 21.90167 |
| rs12691058 | epilepsy | 16 | 7232876 | T | C | 0.5929 | -0.02499 | 0.005654 | 9.88E-06 | 0.000301 | 19.53575 |
| rs7208909 | epilepsy | 17 | 65833546 | T | C | 0.7944 | -0.03222 | 0.006864 | 2.69E-06 | 0.000339 | 22.02356 |
| rs72835272 | epilepsy | 17 | 8194318 | A | G | 0.502 | -0.03014 | 0.005616 | 8.02E-08 | 0.000454 | 28.80374 |
| rs75280876 | epilepsy | 18 | 59274578 | A | T | 0.9295 | -0.056 | 0.010872 | 2.59E-07 | 0.000411 | 26.53196 |
| rs6100474 | epilepsy | 20 | 58092596 | T | C | 0.3425 | -0.02711 | 0.00588 | 4.02E-06 | 0.000331 | 21.2515 |
| rs11907928 | epilepsy | 20 | 33848456 | T | G | 0.8314 | 0.037763 | 0.007451 | 4.03E-07 | 0.0004 | 25.68385 |
| rs76464759 | epilepsy | 21 | 25808751 | T | G | 0.9318 | 0.056418 | 0.011041 | 3.22E-07 | 0.000405 | 26.11132 |
| rs1999321 | epilepsy | 21 | 30530233 | A | C | 0.475 | 0.025008 | 0.005552 | 6.68E-06 | 0.000312 | 20.28536 |
| rs13048435 | epilepsy | 21 | 37343254 | T | G | 0.0507 | 0.057003 | 0.01285 | 9.17E-06 | 0.000313 | 19.67749 |
| rs2223269 | epilepsy | 22 | 35030207 | T | C | 0.2285 | 0.030935 | 0.006609 | 2.86E-06 | 0.000337 | 21.91112 |

Chr, chromosome; EAF, Effect allele frequency; Pos, position; SE, standard error; SNP, single nucleotide polymorphism.

**Table S2.** Characteristics of selected SNPs for focal epilepsy.

| **SNP** | **Trait** | **Chr** | **Pos** | **Effect allele** | **Other allele** | **EAF** | **Beta** | **SE** | ***P value*** | **R^2^** | **F statistic** |
| --- | --- | --- | --- | --- | --- | --- | --- | --- | --- | --- | --- |
| rs1525538 | focal epilepsy | 7 | 1.49E+08 | T | G | 0.7651 | -0.03859 | 0.007995 | 1.39E-06 | 0.000535 | 23.29886 |
| rs2753616 | focal epilepsy | 14 | 86073892 | A | G | 0.5799 | -0.03361 | 0.006861 | 9.69E-07 | 0.00055 | 23.98927 |
| rs7610619 | focal epilepsy | 3 | 39186200 | C | G | 0.9184 | -0.05689 | 0.012367 | 4.23E-06 | 0.000485 | 21.159 |
| rs10246978 | focal epilepsy | 7 | 78147094 | T | C | 0.5783 | 0.031518 | 0.006843 | 4.10E-06 | 0.000485 | 21.21428 |
| rs73105784 | focal epilepsy | 3 | 70480062 | A | C | 0.0972 | -0.05155 | 0.011441 | 6.61E-06 | 0.000466 | 20.30312 |
| rs7805828 | focal epilepsy | 7 | 22758562 | A | G | 0.4071 | 0.032457 | 0.006877 | 2.35E-06 | 0.000509 | 22.27735 |
| rs116753937 | focal epilepsy | 5 | 1.64E+08 | T | C | 0.0165 | 0.132244 | 0.027753 | 1.89E-06 | 0.000568 | 22.70395 |
| rs62272805 | focal epilepsy | 3 | 1.6E+08 | A | G | 0.9297 | -0.05929 | 0.013235 | 7.48E-06 | 0.00046 | 20.06946 |
| rs835760 | focal epilepsy | 11 | 44893024 | T | C | 0.2348 | 0.036094 | 0.008066 | 7.62E-06 | 0.000468 | 20.02468 |
| rs2302756 | focal epilepsy | 13 | 1.14E+08 | A | G | 0.7602 | 0.038074 | 0.007915 | 1.51E-06 | 0.000529 | 23.13504 |
| rs112153128 | focal epilepsy | 2 | 18901976 | C | G | 0.8758 | 0.049631 | 0.010376 | 1.73E-06 | 0.000536 | 22.87598 |
| rs16916972 | focal epilepsy | 8 | 95686214 | T | G | 0.0425 | 0.077569 | 0.017272 | 7.09E-06 | 0.00049 | 20.16808 |
| rs10833113 | focal epilepsy | 11 | 19476053 | A | G | 0.5915 | 0.034313 | 0.006963 | 8.31E-07 | 0.000569 | 24.28402 |
| rs1409032 | focal epilepsy | 13 | 24686133 | A | G | 0.2997 | 0.033486 | 0.007448 | 6.92E-06 | 0.000471 | 20.21311 |
| rs11080800 | focal epilepsy | 18 | 1505023 | T | C | 0.1158 | 0.047251 | 0.010645 | 9.04E-06 | 0.000457 | 19.70379 |
| rs112518225 | focal epilepsy | 16 | 50145180 | A | T | 0.0614 | -0.06334 | 0.014224 | 8.45E-06 | 0.000462 | 19.82825 |
| rs12465710 | focal epilepsy | 2 | 2.07E+08 | T | C | 0.3088 | 0.034253 | 0.007682 | 8.25E-06 | 0.000501 | 19.88168 |
| rs9525161 | focal epilepsy | 13 | 96886373 | A | G | 0.4571 | -0.03259 | 0.006796 | 1.63E-06 | 0.000527 | 22.99102 |
| rs2470565 | focal epilepsy | 3 | 11919651 | T | C | 0.1909 | 0.042426 | 0.008658 | 9.59E-07 | 0.000556 | 24.00888 |
| rs76441232 | focal epilepsy | 3 | 85329725 | A | G | 0.0725 | -0.05785 | 0.013044 | 9.22E-06 | 0.00045 | 19.66835 |
| rs7849871 | focal epilepsy | 9 | 80813747 | T | C | 0.2271 | -0.03857 | 0.008077 | 1.79E-06 | 0.000522 | 22.8091 |
| rs35416677 | focal epilepsy | 2 | 2.4E+08 | A | C | 0.8968 | -0.05214 | 0.011201 | 3.23E-06 | 0.000503 | 21.66805 |
| rs7171131 | focal epilepsy | 15 | 92504340 | T | C | 0.6711 | -0.033 | 0.007212 | 4.75E-06 | 0.000481 | 20.93887 |
| rs2147676 | focal epilepsy | 13 | 53286126 | A | G | 0.5258 | 0.032611 | 0.006778 | 1.50E-06 | 0.00053 | 23.14467 |
| rs7568090 | focal epilepsy | 2 | 1.67E+08 | A | C | 0.768 | -0.03917 | 0.008022 | 1.05E-06 | 0.000547 | 23.84259 |
| rs61580908 | focal epilepsy | 16 | 77334604 | T | C | 0.8795 | 0.050896 | 0.01043 | 1.06E-06 | 0.000549 | 23.81328 |
| rs7313956 | focal epilepsy | 12 | 1.33E+08 | A | C | 0.1936 | -0.04096 | 0.008586 | 1.84E-06 | 0.000524 | 22.76135 |
| rs4979006 | focal epilepsy | 9 | 1.14E+08 | T | C | 0.2896 | 0.038336 | 0.007471 | 2.88E-07 | 0.000605 | 26.32594 |
| rs690254 | focal epilepsy | 7 | 1.46E+08 | T | C | 0.8006 | -0.03866 | 0.00847 | 5.01E-06 | 0.000477 | 20.82918 |
| rs143770404 | focal epilepsy | 15 | 91723744 | A | G | 0.2286 | -0.03796 | 0.008155 | 3.26E-06 | 0.000508 | 21.65866 |
| rs4851251 | focal epilepsy | 2 | 1.01E+08 | T | C | 0.2649 | 0.035395 | 0.007674 | 4.00E-06 | 0.000488 | 21.26962 |
| rs62343369 | focal epilepsy | 5 | 9178683 | T | C | 0.1592 | 0.044374 | 0.009268 | 1.68E-06 | 0.000527 | 22.92391 |
| rs12998431 | focal epilepsy | 2 | 1.9E+08 | A | G | 0.3417 | 0.032447 | 0.007188 | 6.35E-06 | 0.000474 | 20.3753 |
| rs10040295 | focal epilepsy | 5 | 1.18E+08 | A | G | 0.7622 | -0.03607 | 0.007985 | 6.28E-06 | 0.000472 | 20.40238 |
| rs512583 | focal epilepsy | 13 | 30518125 | A | G | 0.8074 | 0.04002 | 0.00857 | 3.02E-06 | 0.000498 | 21.80788 |
| rs502864 | focal epilepsy | 10 | 99764930 | T | C | 0.3081 | 0.033551 | 0.007332 | 4.73E-06 | 0.00048 | 20.93888 |
| rs9387358 | focal epilepsy | 6 | 1.16E+08 | T | C | 0.701 | -0.03284 | 0.007391 | 8.86E-06 | 0.000452 | 19.7394 |
| rs7738855 | focal epilepsy | 6 | 2525055 | T | C | 0.9094 | 0.062442 | 0.011835 | 1.32E-07 | 0.000642 | 27.83491 |
| rs2832038 | focal epilepsy | 21 | 30115325 | A | G | 0.2313 | 0.036379 | 0.008015 | 5.66E-06 | 0.000471 | 20.60162 |
| rs4732135 | focal epilepsy | 7 | 1.35E+08 | A | G | 0.4304 | -0.03227 | 0.006853 | 2.49E-06 | 0.000511 | 22.17368 |
| rs12031954 | focal epilepsy | 1 | 71733081 | T | G | 0.0321 | -0.0895 | 0.019928 | 7.10E-06 | 0.000498 | 20.16811 |
| rs28612129 | focal epilepsy | 12 | 1.14E+08 | A | T | 0.9715 | -0.10031 | 0.020807 | 1.43E-06 | 0.000557 | 23.24077 |
| rs11134138 | focal epilepsy | 5 | 6093701 | T | C | 0.4559 | -0.03496 | 0.006798 | 2.71E-07 | 0.000606 | 26.44926 |
| rs1991912 | focal epilepsy | 2 | 80799512 | T | C | 0.3856 | 0.031404 | 0.006962 | 6.46E-06 | 0.000467 | 20.34824 |
| rs2534590 | focal epilepsy | 7 | 38387731 | T | C | 0.6778 | 0.033348 | 0.007323 | 5.25E-06 | 0.000486 | 20.73797 |
| rs6541281 | focal epilepsy | 1 | 2.32E+08 | T | C | 0.4688 | 0.032236 | 0.006859 | 2.61E-06 | 0.000518 | 22.08902 |

Chr, chromosome; EAF, Effect allele frequency; Pos, position; SE, standard error; SNP, single nucleotide polymorphism.

**Table S3.** Characteristics of selected SNPs for generalized epilepsy.

| **SNP** | **Trait** | **Chr** | **Pos** | **Effect allele** | **Other allele** | **EAF** | **Beta** | **SE** | ***P value*** | **R^2^** | **F statistic** |
| --- | --- | --- | --- | --- | --- | --- | --- | --- | --- | --- | --- |
| rs6672010 | generalized epilepsy | 1 | 1.54E+08 | T | C | 0.3087 | -0.04473 | 0.010014 | 7.94E-06 | 0.000854 | 19.95234 |
| rs10863593 | generalized epilepsy | 1 | 2.08E+08 | T | C | 0.6868 | -0.04564 | 0.009978 | 4.79E-06 | 0.000896 | 20.91968 |
| rs10920265 | generalized epilepsy | 1 | 2.02E+08 | T | C | 0.2051 | -0.05581 | 0.011491 | 1.19E-06 | 0.001016 | 23.58846 |
| rs12137398 | generalized epilepsy | 1 | 7790548 | T | C | 0.1937 | -0.0561 | 0.011776 | 1.90E-06 | 0.000983 | 22.69375 |
| rs72661446 | generalized epilepsy | 1 | 23740708 | A | C | 0.1328 | -0.06246 | 0.013751 | 5.58E-06 | 0.000899 | 20.628 |
| rs876793 | generalized epilepsy | 1 | 2.38E+08 | T | C | 0.6563 | -0.06038 | 0.009847 | 8.67E-10 | 0.001645 | 37.59816 |
| rs2412215 | generalized epilepsy | 1 | 7089050 | C | G | 0.4644 | -0.04998 | 0.009277 | 7.13E-08 | 0.001243 | 29.02806 |
| rs3121733 | generalized epilepsy | 1 | 45736852 | A | T | 0.7948 | 0.0516 | 0.011544 | 7.81E-06 | 0.000868 | 19.97917 |
| rs2493254 | generalized epilepsy | 1 | 3380557 | T | C | 0.0673 | -0.08504 | 0.018498 | 4.29E-06 | 0.000908 | 21.1306 |
| rs1346614 | generalized epilepsy | 2 | 1.55E+08 | T | G | 0.6009 | 0.045173 | 0.009575 | 2.38E-06 | 0.000979 | 22.25761 |
| rs2176263 | generalized epilepsy | 2 | 24092773 | C | G | 0.5987 | -0.04478 | 0.009454 | 2.17E-06 | 0.000964 | 22.43722 |
| rs11677484 | generalized epilepsy | 2 | 1.92E+08 | T | G | 0.2643 | 0.064005 | 0.0105 | 1.09E-09 | 0.001593 | 37.15808 |
| rs4675351 | generalized epilepsy | 2 | 2.04E+08 | T | C | 0.6979 | -0.04558 | 0.010071 | 6.00E-06 | 0.000876 | 20.4829 |
| rs2945452 | generalized epilepsy | 2 | 1.04E+08 | A | T | 0.4955 | 0.065373 | 0.009312 | 2.22E-12 | 0.002137 | 49.27609 |
| rs11890028 | generalized epilepsy | 2 | 1.67E+08 | T | G | 0.7165 | 0.055681 | 0.010289 | 6.23E-08 | 0.00126 | 29.28723 |
| rs11688767 | generalized epilepsy | 2 | 57988194 | A | T | 0.5152 | 0.083722 | 0.009256 | 1.49E-19 | 0.003501 | 81.80508 |
| rs10498240 | generalized epilepsy | 2 | 2.31E+08 | A | C | 0.3271 | -0.04575 | 0.009896 | 3.78E-06 | 0.000921 | 21.37032 |
| rs75900178 | generalized epilepsy | 2 | 1.21E+08 | T | C | 0.0375 | 0.129392 | 0.024571 | 1.39E-07 | 0.001209 | 27.72834 |
| rs17243029 | generalized epilepsy | 3 | 36290458 | A | G | 0.5052 | 0.059171 | 0.00928 | 1.82E-10 | 0.00175 | 40.64992 |
| rs6807049 | generalized epilepsy | 3 | 1.81E+08 | T | G | 0.2819 | -0.05049 | 0.010284 | 9.11E-07 | 0.001032 | 24.10601 |
| rs1392259 | generalized epilepsy | 3 | 1.15E+08 | A | G | 0.5836 | -0.04539 | 0.009409 | 1.41E-06 | 0.001001 | 23.26896 |
| rs12638247 | generalized epilepsy | 3 | 20101137 | T | C | 0.2122 | -0.05312 | 0.011359 | 2.91E-06 | 0.000944 | 21.87244 |
| rs1491686 | generalized epilepsy | 3 | 70092287 | A | G | 0.4548 | 0.045567 | 0.009303 | 9.67E-07 | 0.00103 | 23.9883 |
| rs739431 | generalized epilepsy | 3 | 50255305 | A | G | 0.8588 | 0.085752 | 0.013283 | 1.08E-10 | 0.001783 | 41.67636 |
| rs11720653 | generalized epilepsy | 3 | 17439510 | T | C | 0.6909 | 0.054106 | 0.010134 | 9.36E-08 | 0.00125 | 28.50244 |
| rs11721205 | generalized epilepsy | 3 | 1.79E+08 | T | C | 0.8503 | -0.05773 | 0.012979 | 8.67E-06 | 0.000849 | 19.78299 |
| rs1463849 | generalized epilepsy | 4 | 31148846 | A | G | 0.5947 | -0.06112 | 0.009439 | 9.51E-11 | 0.001801 | 41.92197 |
| rs59588638 | generalized epilepsy | 4 | 3233614 | T | C | 0.9004 | 0.086325 | 0.015476 | 2.43E-08 | 0.001337 | 31.11138 |
| rs11943905 | generalized epilepsy | 4 | 46397617 | T | C | 0.2748 | 0.055441 | 0.010408 | 9.96E-08 | 0.001225 | 28.37448 |
| rs4596374 | generalized epilepsy | 5 | 1.14E+08 | T | C | 0.5355 | -0.06521 | 0.009303 | 2.38E-12 | 0.002116 | 49.1358 |
| rs12659394 | generalized epilepsy | 5 | 19960174 | T | C | 0.5616 | 0.043072 | 0.009323 | 3.84E-06 | 0.000914 | 21.34256 |
| rs2365763 | generalized epilepsy | 5 | 88980640 | T | G | 0.9065 | 0.0761 | 0.015964 | 1.87E-06 | 0.000982 | 22.72233 |
| rs419677 | generalized epilepsy | 5 | 1.09E+08 | A | G | 0.7112 | 0.050401 | 0.010225 | 8.28E-07 | 0.001043 | 24.29296 |
| rs7705607 | generalized epilepsy | 5 | 78609713 | A | G | 0.3995 | -0.04254 | 0.009451 | 6.78E-06 | 0.000868 | 20.25725 |
| rs1369126 | generalized epilepsy | 5 | 1.37E+08 | C | G | 0.4688 | -0.05917 | 0.00928 | 1.82E-10 | 0.001744 | 40.64983 |
| rs11740812 | generalized epilepsy | 5 | 1.75E+08 | A | G | 0.5909 | -0.04709 | 0.009468 | 6.57E-07 | 0.001072 | 24.73853 |
| rs9401560 | generalized epilepsy | 6 | 98534495 | C | G | 0.959 | -0.11421 | 0.023535 | 1.22E-06 | 0.001026 | 23.54949 |
| rs588277 | generalized epilepsy | 6 | 1.58E+08 | T | C | 0.2999 | -0.04905 | 0.010176 | 1.43E-06 | 0.00101 | 23.23033 |
| rs4052834 | generalized epilepsy | 6 | 16974460 | A | G | 0.7892 | 0.061667 | 0.01135 | 5.54E-08 | 0.001265 | 29.51493 |
| rs2350523 | generalized epilepsy | 6 | 65833692 | C | G | 0.7274 | 0.051846 | 0.010411 | 6.37E-07 | 0.001066 | 24.79828 |
| rs13219424 | generalized epilepsy | 6 | 1.28E+08 | T | C | 0.3042 | -0.05223 | 0.010152 | 2.68E-07 | 0.001155 | 26.46876 |
| rs11156380 | generalized epilepsy | 6 | 1.05E+08 | T | G | 0.2639 | -0.04697 | 0.010538 | 8.30E-06 | 0.000857 | 19.86315 |
| rs2077537 | generalized epilepsy | 6 | 31606522 | C | G | 0.197 | -0.05515 | 0.01164 | 2.16E-06 | 0.000962 | 22.44675 |
| rs117773972 | generalized epilepsy | 7 | 20388083 | T | C | 0.0149 | 0.179171 | 0.03859 | 3.43E-06 | 0.000942 | 21.5555 |
| rs2012593 | generalized epilepsy | 7 | 78014174 | T | C | 0.3352 | -0.04368 | 0.009797 | 8.28E-06 | 0.00085 | 19.87202 |
| rs7811417 | generalized epilepsy | 7 | 21534152 | T | C | 0.3369 | 0.044456 | 0.009801 | 5.72E-06 | 0.000883 | 20.57348 |
| rs7810452 | generalized epilepsy | 7 | 86034853 | C | G | 0.4491 | 0.04503 | 0.009298 | 1.28E-06 | 0.001003 | 23.4526 |
| rs37276 | generalized epilepsy | 7 | 41343524 | T | G | 0.2392 | -0.06473 | 0.010984 | 3.78E-09 | 0.001525 | 34.72438 |
| rs77555604 | generalized epilepsy | 8 | 89353507 | A | G | 0.888 | -0.0654 | 0.014683 | 8.42E-06 | 0.000851 | 19.83639 |
| rs6999315 | generalized epilepsy | 8 | 18795045 | A | G | 0.8044 | 0.055204 | 0.011733 | 2.54E-06 | 0.000959 | 22.13508 |
| rs1467048 | generalized epilepsy | 8 | 1.31E+08 | A | G | 0.1793 | -0.05728 | 0.012106 | 2.23E-06 | 0.000966 | 22.38991 |
| rs13301660 | generalized epilepsy | 9 | 1.39E+08 | T | C | 0.267 | -0.04827 | 0.010478 | 4.09E-06 | 0.000912 | 21.22266 |
| rs10811199 | generalized epilepsy | 9 | 19532826 | T | G | 0.8035 | -0.05312 | 0.011659 | 5.22E-06 | 0.000891 | 20.75536 |
| rs13294415 | generalized epilepsy | 9 | 77669152 | C | G | 0.2142 | -0.05619 | 0.01135 | 7.38E-07 | 0.001063 | 24.51024 |
| rs295268 | generalized epilepsy | 9 | 86429305 | T | C | 0.7369 | 0.07284 | 0.010515 | 4.30E-12 | 0.002057 | 47.9792 |
| rs1410058 | generalized epilepsy | 10 | 97133863 | A | G | 0.4674 | 0.049132 | 0.009289 | 1.23E-07 | 0.001202 | 27.97114 |
| rs4443967 | generalized epilepsy | 10 | 18581361 | T | C | 0.3405 | 0.044975 | 0.009883 | 5.33E-06 | 0.000908 | 20.70974 |
| rs3099370 | generalized epilepsy | 10 | 61696597 | A | G | 0.5919 | 0.041991 | 0.009419 | 8.28E-06 | 0.000852 | 19.8721 |
| rs3740422 | generalized epilepsy | 10 | 1.04E+08 | C | G | 0.3428 | 0.071174 | 0.009762 | 3.07E-13 | 0.002282 | 53.15405 |
| rs10741221 | generalized epilepsy | 10 | 1.33E+08 | T | C | 0.5449 | -0.05197 | 0.009303 | 2.32E-08 | 0.001339 | 31.2007 |
| rs1982350 | generalized epilepsy | 11 | 13350131 | A | G | 0.3804 | 0.04265 | 0.009548 | 7.94E-06 | 0.000857 | 19.95241 |
| rs2072114 | generalized epilepsy | 11 | 61605215 | A | G | 0.8747 | 0.067477 | 0.014 | 1.43E-06 | 0.000998 | 23.23039 |
| rs1625595 | generalized epilepsy | 11 | 66078129 | T | C | 0.4774 | 0.041586 | 0.009351 | 8.71E-06 | 0.000863 | 19.77403 |
| rs74651462 | generalized epilepsy | 11 | 99578804 | A | G | 0.0595 | 0.08969 | 0.019596 | 4.72E-06 | 0.0009 | 20.94714 |
| rs11065302 | generalized epilepsy | 12 | 1.21E+08 | T | C | 0.088 | 0.076282 | 0.016345 | 3.05E-06 | 0.000934 | 21.77902 |
| rs73177990 | generalized epilepsy | 12 | 1.05E+08 | T | C | 0.9286 | -0.08844 | 0.018039 | 9.42E-07 | 0.001037 | 24.03735 |
| rs12832640 | generalized epilepsy | 12 | 81578851 | A | T | 0.474 | -0.05054 | 0.009269 | 4.97E-08 | 0.001273 | 29.72176 |
| rs7134809 | generalized epilepsy | 12 | 1.33E+08 | T | C | 0.8372 | 0.064881 | 0.01261 | 2.67E-07 | 0.001147 | 26.46874 |
| rs4762030 | generalized epilepsy | 12 | 52323461 | T | G | 0.0214 | 0.199289 | 0.03231 | 6.90E-10 | 0.001663 | 38.04102 |
| rs9301451 | generalized epilepsy | 13 | 89324988 | T | C | 0.3612 | -0.04795 | 0.009668 | 7.05E-07 | 0.001061 | 24.5995 |
| rs9581702 | generalized epilepsy | 13 | 27131688 | T | C | 0.3081 | -0.04855 | 0.010026 | 1.28E-06 | 0.001005 | 23.45265 |
| rs2215084 | generalized epilepsy | 15 | 92692946 | A | G | 0.5419 | -0.04127 | 0.009331 | 9.74E-06 | 0.000846 | 19.56124 |
| rs191961842 | generalized epilepsy | 15 | 41005998 | T | G | 0.9854 | -0.17599 | 0.038645 | 5.27E-06 | 0.000891 | 20.7371 |
| rs7163861 | generalized epilepsy | 15 | 61632785 | A | G | 0.7343 | 0.049254 | 0.010506 | 2.77E-06 | 0.000947 | 21.97549 |
| rs3131574 | generalized epilepsy | 15 | 50740939 | A | C | 0.4638 | 0.047295 | 0.009292 | 3.57E-07 | 0.001113 | 25.90587 |
| rs62014006 | generalized epilepsy | 16 | 7344941 | T | G | 0.4627 | 0.05501 | 0.009322 | 3.61E-09 | 0.001505 | 34.81881 |
| rs6565217 | generalized epilepsy | 16 | 31083324 | A | G | 0.3797 | 0.043252 | 0.009548 | 5.90E-06 | 0.000881 | 20.51914 |
| rs62037373 | generalized epilepsy | 16 | 28893295 | C | G | 0.3707 | 0.049706 | 0.009672 | 2.77E-07 | 0.001153 | 26.40697 |
| rs12451882 | generalized epilepsy | 17 | 42301922 | T | C | 0.3165 | -0.04492 | 0.00996 | 6.49E-06 | 0.000873 | 20.33839 |
| rs16955463 | generalized epilepsy | 17 | 46399497 | T | G | 0.2627 | -0.06473 | 0.010516 | 7.47E-10 | 0.001623 | 37.89305 |
| rs755046 | generalized epilepsy | 17 | 48610272 | C | G | 0.7195 | 0.048363 | 0.010308 | 2.71E-06 | 0.000944 | 22.01293 |
| rs4123417 | generalized epilepsy | 17 | 18715642 | T | C | 0.514 | 0.048241 | 0.009306 | 2.17E-07 | 0.001163 | 26.87149 |
| rs2585398 | generalized epilepsy | 17 | 8054860 | A | C | 0.5432 | -0.05826 | 0.009405 | 5.84E-10 | 0.001685 | 38.37461 |
| rs1124980 | generalized epilepsy | 18 | 67548627 | A | G | 0.5956 | -0.04206 | 0.009468 | 8.91E-06 | 0.000852 | 19.72965 |
| rs9949868 | generalized epilepsy | 18 | 29727583 | A | G | 0.4607 | -0.04332 | 0.009356 | 3.66E-06 | 0.000932 | 21.43505 |
| rs75483641 | generalized epilepsy | 19 | 2123928 | T | C | 0.1516 | -0.08456 | 0.012965 | 6.93E-11 | 0.001839 | 42.53276 |
| rs2833089 | generalized epilepsy | 21 | 32165561 | A | G | 0.3715 | -0.06499 | 0.009582 | 1.18E-11 | 0.001972 | 45.99156 |
| rs2836950 | generalized epilepsy | 21 | 40604429 | C | G | 0.6366 | 0.048452 | 0.009681 | 5.58E-07 | 0.001086 | 25.0479 |
| rs11701610 | generalized epilepsy | 21 | 21677669 | A | T | 0.5975 | 0.055753 | 0.00948 | 4.08E-09 | 0.001495 | 34.58321 |
| rs35662099 | generalized epilepsy | 22 | 38053909 | T | C | 0.4191 | -0.04606 | 0.009418 | 1.01E-06 | 0.001033 | 23.91003 |
| rs5761202 | generalized epilepsy | 22 | 26191160 | T | C | 0.7146 | -0.0479 | 0.010263 | 3.05E-06 | 0.000936 | 21.77899 |
| rs469999 | generalized epilepsy | 22 | 48637056 | A | G | 0.3188 | -0.06275 | 0.010002 | 3.52E-10 | 0.00171 | 39.3597 |
| rs130539 | generalized epilepsy | 22 | 33176450 | T | C | 0.8819 | 0.064776 | 0.014436 | 7.21E-06 | 0.000874 | 20.1314 |

Chr, chromosome; EAF, Effect allele frequency; Pos, position; SE, standard error; SNP, single nucleotide polymorphism.

| **Table S4.** Characteristics of selected SNPs for ALS. | | | | | | | | | | | |
| --- | --- | --- | --- | --- | --- | --- | --- | --- | --- | --- | --- |
| **SNP** | **Trait** | **Chr** | **Pos.** | **Effect allele** | **Other allele** | **EAF** | **Beta** | **SE** | ***P value*** | **R^2^** | **F statistic** |
| rs7552104 | ALS | 1 | 94213626 | C | T | 0.1701 | -0.089 | 0.0182 | 1.06E-06 | 0.002236352 | 180.6718767 |
| rs2442404 | ALS | 1 | 204905020 | A | T | 0.508 | -0.0635 | 0.0142 | 7.22E-06 | 0.002015609 | 162.8023458 |
| rs72714928 | ALS | 1 | 161207232 | C | T | 0.162 | 0.0869 | 0.0183 | 2.12E-06 | 0.002050353 | 165.6144011 |
| rs17302583 | ALS | 1 | 181029711 | A | G | 0.0335 | -0.1896 | 0.0422 | 6.93E-06 | 0.002327841 | 188.0804347 |
| rs146107389 | ALS | 2 | 144648691 | C | T | 0.0146 | 0.3509 | 0.0764 | 4.39E-06 | 0.003542927 | 286.603637 |
| rs61527579 | ALS | 2 | 76237892 | C | T | 0.1563 | -0.0906 | 0.0193 | 2.67E-06 | 0.002164878 | 174.8850871 |
| rs150628303 | ALS | 3 | 49955296 | G | A | 0.0553 | 0.1478 | 0.0329 | 7.05E-06 | 0.002282432 | 184.4031931 |
| rs74856549 | ALS | 3 | 150697363 | G | T | 0.0136 | 0.5168 | 0.1143 | 6.18E-06 | 0.007165838 | 581.7928922 |
| rs2013478 | ALS | 4 | 170602223 | T | C | 0.3391 | 0.0656 | 0.0144 | 4.94E-06 | 0.001928862 | 155.7822116 |
| rs2239727 | ALS | 4 | 2822289 | A | C | 0.5443 | -0.0621 | 0.014 | 9.21E-06 | 0.001913069 | 154.5042164 |
| rs112913348 | ALS | 5 | 107889888 | T | C | 0.0666 | -0.1277 | 0.0282 | 5.84E-06 | 0.002027467 | 163.7620904 |
| rs10463311 | ALS | 5 | 150410835 | T | C | 0.7441 | -0.0854 | 0.0156 | 4.00E-08 | 0.002777457 | 224.5088089 |
| rs538622 | ALS | 5 | 172347679 | G | A | 0.3962 | -0.0693 | 0.0139 | 6.69E-07 | 0.002297757 | 185.6441387 |
| rs34182166 | ALS | 5 | 150582494 | G | A | 0.0611 | 0.1372 | 0.03 | 4.70E-06 | 0.002159727 | 174.4680412 |
| rs9364669 | ALS | 6 | 163071272 | T | C | 0.0488 | 0.1397 | 0.0315 | 9.20E-06 | 0.001811818 | 146.312083 |
| rs9379980 | ALS | 6 | 27306518 | T | C | 0.5841 | 0.0619 | 0.0139 | 8.26E-06 | 0.001861605 | 150.3400894 |
| rs2295767 | ALS | 6 | 2954557 | T | C | 0.1615 | -0.0844 | 0.019 | 8.75E-06 | 0.001929259 | 155.8142968 |
| rs77041237 | ALS | 7 | 18420247 | A | G | 0.0236 | 0.2259 | 0.0507 | 8.42E-06 | 0.00235181 | 190.0215945 |
| rs118049474 | ALS | 7 | 42430472 | C | T | 0.0655 | -0.1335 | 0.0284 | 2.66E-06 | 0.002181791 | 176.2543537 |
| rs17148125 | ALS | 7 | 124926381 | C | G | 0.1853 | 0.0851 | 0.0181 | 2.66E-06 | 0.002186564 | 176.6408109 |
| rs17070492 | ALS | 8 | 2420855 | G | C | 0.1015 | -0.1242 | 0.0234 | 1.04E-07 | 0.002813567 | 227.4359409 |
| rs11786739 | ALS | 8 | 145042001 | G | A | 0.0584 | 0.1371 | 0.029 | 2.20E-06 | 0.002067208 | 166.9786918 |
| rs78157304 | ALS | 9 | 71612479 | G | C | 0.054 | 0.1405 | 0.0318 | 9.90E-06 | 0.002016822 | 162.900518 |
| rs3849943 | ALS | 9 | 27543382 | T | C | 0.7518 | -0.1764 | 0.0155 | 3.77E-30 | 0.011612648 | 947.0703106 |
| rs16926548 | ALS | 10 | 71405197 | G | T | 0.1203 | 0.0931 | 0.021 | 9.60E-06 | 0.00183455 | 148.1512054 |
| rs11195948 | ALS | 10 | 114163515 | T | C | 0.346 | -0.0707 | 0.0144 | 9.14E-07 | 0.002262157 | 182.7613558 |
| rs117860708 | ALS | 11 | 1558447 | A | G | 0.0302 | 0.2041 | 0.0451 | 5.93E-06 | 0.002440086 | 197.1715654 |
| rs79735246 | ALS | 11 | 117559725 | G | A | 0.0582 | -0.138 | 0.0312 | 9.95E-06 | 0.002087708 | 168.638066 |
| rs61880881 | ALS | 11 | 22270782 | C | A | 0.0664 | 0.1317 | 0.0281 | 2.73E-06 | 0.002150456 | 173.7174919 |
| rs7947433 | ALS | 11 | 35216814 | G | A | 0.5607 | 0.0639 | 0.0142 | 6.69E-06 | 0.002011516 | 162.4710874 |
| rs117704471 | ALS | 12 | 12904565 | A | G | 0.019 | 0.3336 | 0.0712 | 2.82E-06 | 0.00414863 | 335.8058894 |
| rs142321490 | ALS | 12 | 58676132 | C | G | 0.0183 | 0.3172 | 0.0513 | 6.15E-10 | 0.003615149 | 292.4672649 |
| rs74654358 | ALS | 12 | 64881967 | A | G | 0.0473 | 0.1976 | 0.0337 | 4.66E-09 | 0.003519016 | 284.6625349 |
| rs11842416 | ALS | 13 | 46035870 | C | T | 0.2035 | 0.0814 | 0.0172 | 2.36E-06 | 0.002147974 | 173.5165889 |
| rs10143310 | ALS | 14 | 92540381 | C | G | 0.2374 | 0.0821 | 0.0161 | 3.23E-07 | 0.002440584 | 197.2119372 |
| rs10139154 | ALS | 14 | 31147498 | T | C | 0.465 | 0.0767 | 0.0146 | 1.44E-07 | 0.002927032 | 236.6348267 |
| rs9901522 | ALS | 17 | 14673934 | T | C | 0.0653 | 0.1462 | 0.0273 | 8.59E-08 | 0.002609217 | 210.8739631 |
| rs34517613 | ALS | 17 | 26610252 | T | C | 0.1235 | -0.1057 | 0.0223 | 2.18E-06 | 0.002418794 | 195.446877 |
| rs2285642 | ALS | 17 | 34912744 | C | G | 0.5411 | 0.0674 | 0.0141 | 1.80E-06 | 0.002256033 | 182.2654766 |
| rs28623193 | ALS | 17 | 63966503 | G | A | 0.741 | 0.2081 | 0.044 | 2.21E-06 | 0.016622339 | 1362.542117 |
| rs79068040 | ALS | 18 | 45740166 | T | C | 0.0255 | 0.2058 | 0.0444 | 3.63E-06 | 0.002104955 | 170.0341051 |
| rs12973192 | ALS | 19 | 17753239 | G | C | 0.3247 | 0.1205 | 0.0153 | 3.92E-15 | 0.006367708 | 516.5776073 |
| rs8125607 | ALS | 20 | 48526705 | A | G | 0.3048 | 0.0688 | 0.0151 | 4.92E-06 | 0.002006003 | 162.0249198 |
| rs75087725 | ALS | 21 | 45753117 | A | C | 0.0153 | 0.5145 | 0.0672 | 1.85E-14 | 0.007976202 | 648.1151561 |
| rs67710834 | ALS | 22 | 50742346 | C | G | 0.4018 | 0.0698 | 0.015 | 3.31E-06 | 0.002342055 | 189.2316005 |

ALS, Amyotrophic lateral sclerosis; Chr, chromosome; EAF, Effect allele frequency; Pos, position; SE, standard error; SNP, single nucleotide polymorphism.

**Table S5.** The removed SNPs associated with epilepsy and its subtypes from MR analysis

| **Disease** | **Removed SNPs** | **Associated gene** |
| --- | --- | --- |
| epilepsy | rs9817162 | GAP43 |
|  | rs3740422 | O-GlcNAcase |
|  | rs60055328 | SCN1A |
| generalized epilepsy | rs62014006 | RBFOX1 |
|  | rs1982350 | BMAL1 |
|  | rs12137398 | CAMTA1 |
|  | rs4052834 | ATXN1-AS1 |
|  | rs11677484 | glutaminase |
|  | rs13301660 | SEC16A |
|  | rs37276 | INHBA |
|  | rs13219424 | PTPRK |
|  | rs3740422 | O-GlcNAcase |
|  | rs2072114 | FADS2 |
|  | rs11890028 | SCN1A |
|  | rs1625595 | CD248 |
|  | rs11943905 | GABRA2 |
| focal epilepsy | rs10246978 | MAGI2 |

MR, Mendelian randomization; SNP, single nucleotide polymorphism.

**Table S6.** Reverse causal relationships of ALS with epilepsy performed by MR.

|  | **Outcomes** | | **method** | **nSNP** | **OR** | **95%CI** | ***P*** |
| --- | --- | --- | --- | --- | --- | --- | --- |
| **Primary analysis** |  | |  |  |  |  |  |
|  | epilepsy | | IVW | 20 | 1.005 | 0.971-1.039 | 0.793 |
|  |  | | Weighted median | 20 | 0.998 | 0.951-1.048 | 0.945 |
|  |  | | MR Egger | 20 | 1.009 | 0.908-1.121 | 0.870 |
|  |  | | Simple mode | 20 | 1.012 | 0.930-1.101 | 0.790 |
|  |  | | Weighted mode | 20 | 1.003 | 0.941-1.069 | 0.931 |
|  | generalized epilepsy | | IVW | 20 | 1.003 | 0.926-1.086 | 0.943 |
|  |  | | Weighted median | 20 | 0.989 | 0.902-1.084 | 0.813 |
|  |  | | MR Egger | 20 | 1.069 | 0.830-1.376 | 0.613 |
|  |  | | Simple mode | 20 | 0.990 | 0.836-1.171 | 0.907 |
|  |  | | Weighted mode | 20 | 0.977 | 0.879-1.087 | 0.676 |
|  | focal epilepsy | | IVW | 20 | 1.003 | 0.962-1.045 | 0.903 |
|  |  | | Weighted median | 20 | 0.996 | 0.937-1.059 | 0.894 |
|  |  | | MR Egger | 20 | 0.983 | 0.863-1.120 | 0.798 |
|  |  | | Simple mode | 20 | 0.985 | 0.882-1.101 | 0.798 |
|  |  | | Weighted mode | 20 | 0.991 | 0.914-1.073 | 0.825 |
| **Supplemental analysis** |  | |  |  |  |  |  |
|  | epilepsy | | IVW | 2 | 1.003 | 0.940-1.070 | 0.920 |
|  | generalized epilepsy | | IVW | 2 | 1.011 | 0.839-1.217 | 0.912 |
|  | focal epilepsy | | IVW | 2 | 0.987 | 0.912-1.069 | 0.755 |
|  | | CI: confidence interval; FDR: false discovery rate; IVW: inverse-variance weighted; nSNPs: number of single-nucleotide polymorphisms; OR: odds ratio; ALS: amyotrophic lateral sclerosis. | | | | | |

**Supplemental Figure**

**Figure S1.** The causal effect of generalized epilepsy on ALS risk in supplemental analysis. (A) Scatter plot, (B) Funnel plot, (C) Forest plot, and (D) Leave one out plot. ALS, Amyotrophic lateral sclerosis.


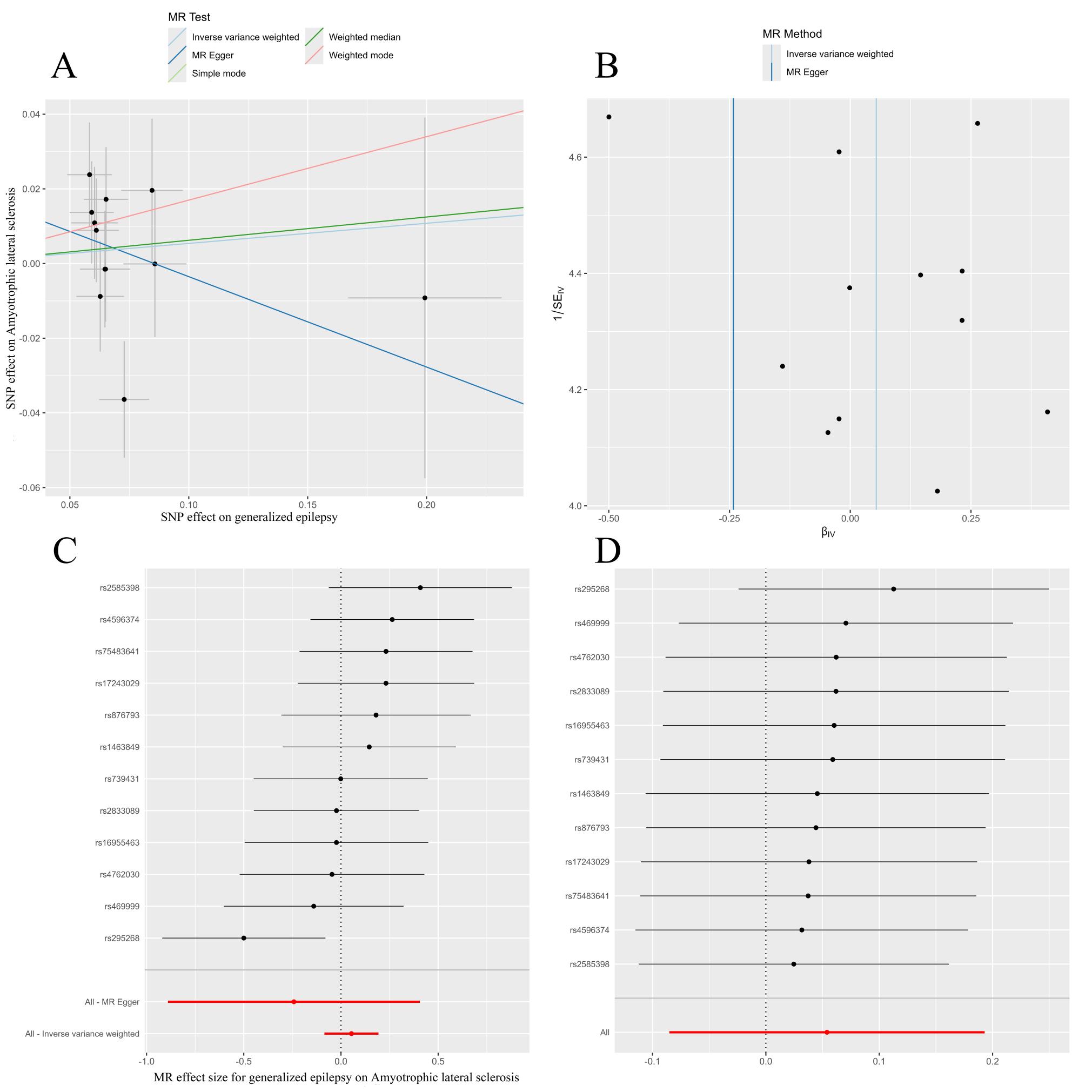


**Figure S2.** The causal effect of ALS on epilepsy risk in primary analysis. (A) Scatter plot, (B) Funnel plot, (C) Forest plot, and (D) Leave one out plot. ALS, Amyotrophic lateral sclerosis.


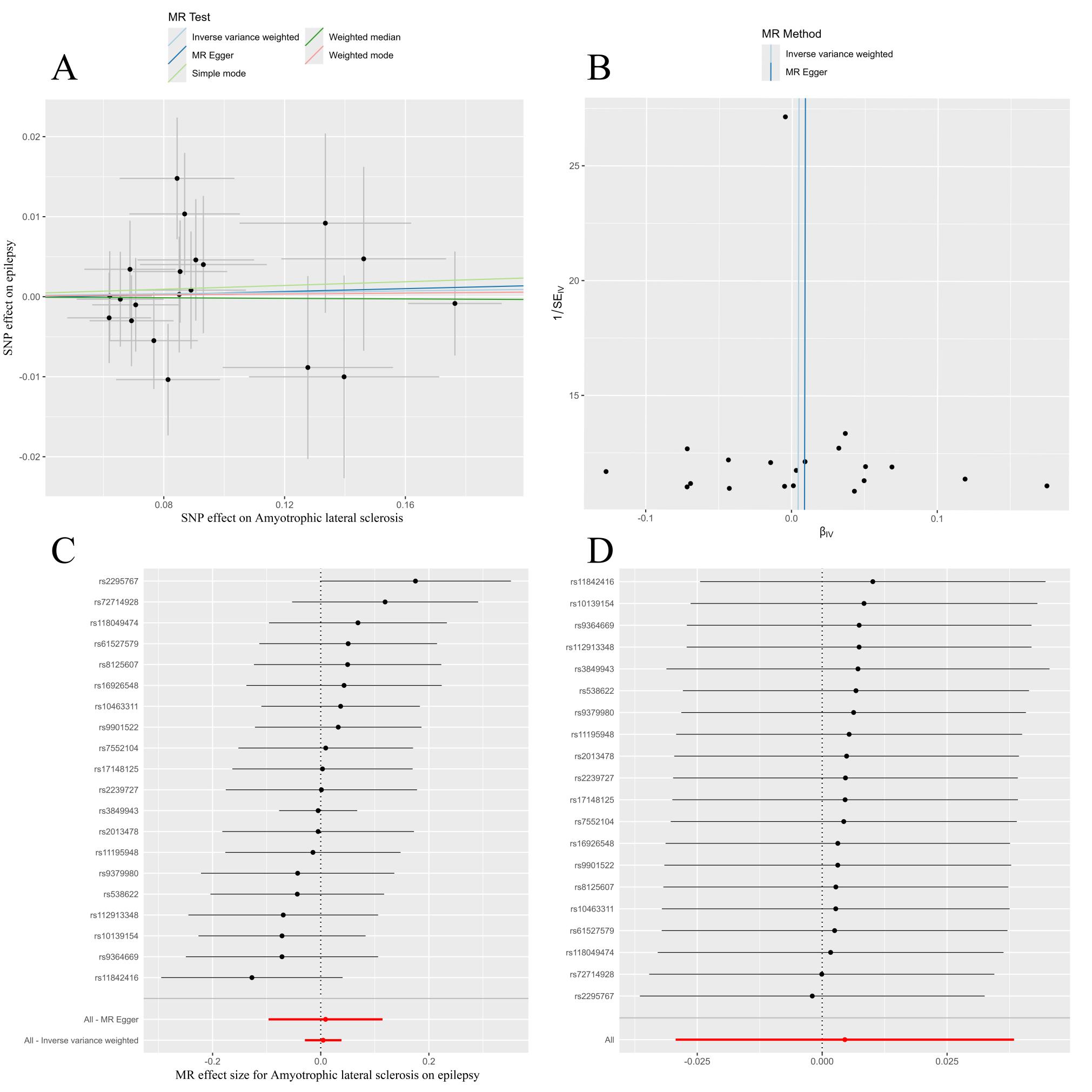


**Figure S3.** The causal effect of ALS on focal epilepsy risk in primary analysis. (A) Scatter plot, (B) Funnel plot, (C) Forest plot, and (D) Leave one out plot. ALS, Amyotrophic lateral sclerosis.


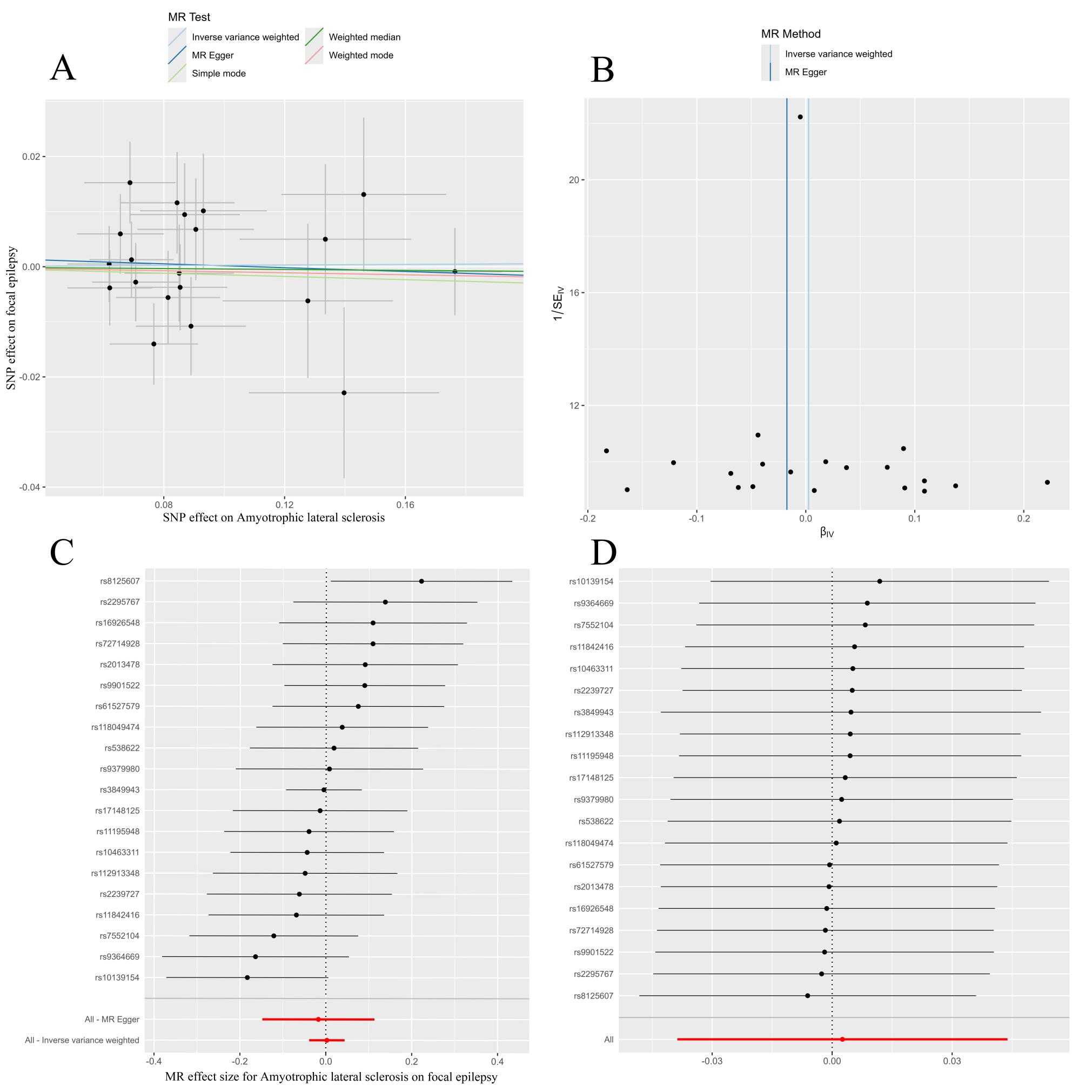


**Figure S4.** The causal effect of ALS on generalized epilepsy risk in primary analysis. (A) Scatter plot, (B) Funnel plot, (C) Forest plot, and (D) Leave one out plot. ALS, Amyotrophic lateral sclerosis.

**
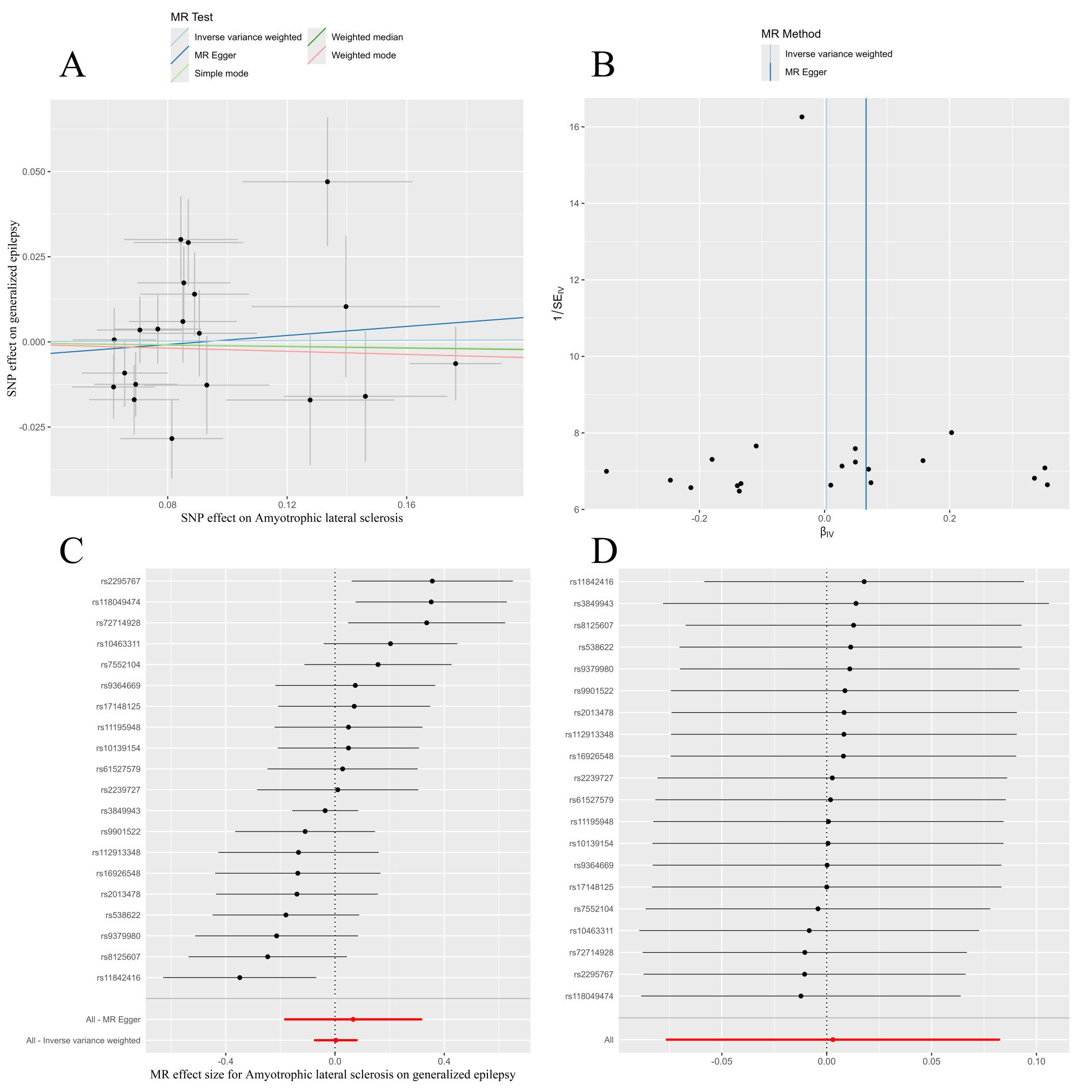
**
